# Novel Organotin(IV) Schiff Base Complexes with Histidine Derivatives: Synthesis, Characterization, and Biological Activity

**DOI:** 10.1155/2013/502713

**Published:** 2013-06-24

**Authors:** Ariadna Garza-Ortiz, Carlos Camacho-Camacho, Teresita Sainz-Espuñes, Irma Rojas-Oviedo, Luis Raúl Gutiérrez-Lucas, Atilano Gutierrez Carrillo, Marco A. Vera Ramirez

**Affiliations:** ^1^Departamento de Sistemas Biológicos, Universidad Autónoma Metropolitana-Unidad Xochimilco, Calzada de Hueso 1100, Colonia Villa Quietud, 04960 Coyoacán, D.F., Mexico; ^2^Departamento de Resonancia Magnética Nuclear, Universidad Autónoma Metropolitana, Unidad Iztapalapa, San Rafael Atlixco, No. 186, Col. Vicentina, 09340 Iztapalapa, D. F., Mexico

## Abstract

Five novel tin Schiff base complexes with histidine analogues (derived from the condensation reaction between L-histidine and 3,5-di-*tert*-butyl-2-hydroxybenzaldehyde) have been synthesized and characterized. Characterization has been completed by IR and high-resolution mass spectroscopy, 1D and 2D solution NMR (^1^H, ^13^C  and ^119^Sn), as well as solid state ^119^Sn NMR. The spectroscopic evidence shows two types of structures: a trigonal bipyramidal stereochemistry with the tin atom coordinated to five donating atoms (two oxygen atoms, one nitrogen atom, and two carbon atoms belonging to the alkyl moieties), where one molecule of ligand is coordinated in a three dentate fashion. The second structure is spectroscopically described as a tetrahedral tin complex with four donating atoms (one oxygen atom coordinated to the metal and three carbon atoms belonging to the alkyl or aryl substituents), with one molecule of ligand attached. The antimicrobial activity of the tin compounds has been tested against the growth of bacteria *in vitro* to assess their bactericidal properties. While pentacoordinated compounds **1**, **2**, and **3** are described as moderate effective to noneffective drugs against both Gram-positive and Gram-negative bacteria, tetracoordinated tin(IV) compounds **4** and **5** are considered as moderate effective and most effective compounds, respectively, against the methicillin-resistant *Staphylococcus aureus* strains (Gram-positive).

## 1. Introduction

The use of metal complexes as chemotherapeutic agents in the treatment of illness, which are a major public health concern, appears as a very attractive alternative. The success of cisplatin for the treatment of testicular and ovarian cancer attracted research attention to other metal-based antineoplastic agents. Metal-based compounds are of particular interest due to their physical and chemical properties. Properties such as ligand exchange rates, redox properties, oxidation states, coordination affinities, solubility, biodisponibility, and biodistribution could be modified in order to increase the therapeutic effect while reducing the side effects.

Although the design of a metal-based compound with good therapeutic index is still rather empirical, a number of potential metal-based bactericide compounds have been fully described in the literature. The evidence about specific or selective bonding of metals and organometallic species to donor sites in biological structures is very limited, so trustable mechanisms of biological activity and valid structure-activity relationships are limited as well. 

One approach that could produce successful results involves the metal coordination of ligands with well-known biological activity. In this way, the designed metal-based compound combines ligands with an important biological activity and pharmacologically active metals in a single chemical moiety. This strategy could produce enhanced efficacy and reduced toxic or side effects while lowering the therapeutic doses and/or overcoming drug resistance mechanisms. Additionally the metal could act as a carrier and/or stabilizer of the drug until it is able to reach the target. At the same time, the organic ligand with well-known biological activity could transport and protect the metal, then avoiding side reactions on its route to the potential targets. The metal-ligand combined effects may result in an important improvement in the activity of the resulting coordination compounds [1, 2].

Amino acids and their derivatives are excellent ligand candidates due to their coordination properties and biological importance. In addition, their solubility properties and specific mechanisms of transport could facilitate their biological uptake. In particular histidine is a very important bioactive amino acid with multiple physiological functions. 

The histidine binding to transition metal ions in biological systems has a major physicochemical role in several proteins, as the X-ray structural determination studies of metalloproteins like carbonic anhydrase, carboxypeptidase, plastocyanin, or azurin among others have demonstrated [3]. Additionally, histidine plays a major role in the zinc metabolism acting as the major zinc binding moiety in serum [4]. 

Schiff bases represent an important group of compounds in organic chemistry because they are starting materials in the synthesis of industrial products where carbon-nitrogen bonds are present [5]. In particular, this N–C bond is involved in several biological functions allowing the Schiff bases to behave, for instance, as antimicrobial, anti-inflamatory, antitumour, or antiviral drugs [6].

The design of organotin derivatives with biologically important ligands, like antibiotics [7, 8], anticancer drugs, [9] and some other biologically relevant substrates [10], has been explored in the past years [11]. Organotin derivatives of amino acids have been of interest as possible biocides [12]; for example, tricyclohexyltin alaninate is a powerful fungicide and bactericide [13]. This compound has been described as a substrate of peptide syntheses as well [14]. Amino acids and dipeptides which are considered potentially polydentate ligands could afford interesting structural possibilities when coordinated to organotin(IV) groups, since organotin(IV) compounds adopt higher coordination numbers whenever favorable conditions exist [15]. 

One of our research goals is the study of organotin(IV) compounds with important biological activity. In this order of ideas we designed, prepared, and characterized diorganotin and triorganotin derivatives of an L-histidine Schiff base analogue, with biological application as antibacterial compounds. The L-histidine derivative was obtained by condensation reaction of L-histidine and 3,5-di-*tert*-butyl-2-hydroxybenzaldehyde. Posterior complexation of the L-histidine analogue was achieved with tin(IV) organometallic reagents. 

To the best of our knowledge there are no reports on organotin(IV) compounds of L-histidine Schiff base derivatives. We have characterized these compounds not only spectroscopically but also with regard to their antibacterial activity against *Escherichia coli*, *Staphylococcus aureus*, *Pseudomona aeruginosa*, *Shigella dysenteriae*, *Salmonella typhimurium*, *Salmonella typhi*, *Proteus mirabilis*, *Klebsiella pneumonia*, and *Serratia marcescens*. The compounds described in this work are all new and some of them showing promising antibacterial activity for methicillin-resistant *Staphylococcus aureus* strains (Gram-positive).

## 2. Experimental

### 2.1. Materials and Methodology

Di-*n*-methyltin oxide, Di-*n*-butyltin oxide, di-*n*-phenyl oxide, bis-*n*-tributyltin oxide, triphenyltin hydroxide, L-histidine 99%, and 3,5-di-*tert*.butyl-2-hydroxybenzaldehyde 99% were purchased from Aldrich and were used without further purification. Solvents were freshly distilled before use following the standard procedures. Elemental analyses were performed in an Eager 300 analyzer. IR spectra were obtained on a Nicolet FT-55X apparatus as KBr discs of each compound. Melting points were measured on a Fisher-Johns melting point apparatus and are uncorrected. ^1^H, ^13^C and ^119^Sn NMR spectra were recorded with a Varian spectrometer operating at 300 MHz using CDCl_3_ or CD_3_OD as solvent and TMS as a reference. Solid state ^119^Sn NMR spectra were recorded with a Bruker AVANCE-II, 300 MHz NMR spectrometer, using a 4 mm CP-MAS probe. ^119^Sn chemical shift referencing is toward tetramethyltin. High-resolution mass spectra were obtained by LC/MSD TOF on an Agilent Technologies instrument with APCI as ionization source.

#### 2.1.1. Synthesis of Tin Derivatives

*Compounds **1**–**5***. To a solution of 3,5-di-*tert*-butyl-2-hydroxybenzaldehyde (2 mmol, 468 mg) in 16 mL of toluene at RT, L-histidine (2 mmol, 310.32 mg), 6 mL of ethanol, and the corresponding tin derivative (2 mmol) were successively added to a flask equipped with a Dean-Stark moisture trap (filled with dry toluene). After 24 h under gentle reflux, the solvents were evaporated giving yellow solids in all cases. Purification was achieved by recrystallization from dichloromethane-hexane (3 : 1) mixtures.

*[(C*_21_ *H*_27_ *N*_3_ *O*_3_ *)Sn(CH*_3_)_2_ *](0.4CH*_2_ *Cl*_2_ *) (**1**)*. A yellow crystalline solid **1** was obtained in moderate yield (350 mg, 33.76%). Elemental analysis for C_23,4_H_33,8_Cl_0,8_N_3_O_3_Sn: Calcd (%): C, 50.90; N, 7.61; H, 6.17. Found (%): C, 50.96; N, 7.27; H, 5.75. IR (KBr disk) cm^−1^: 3445 s, 2958–2869 s, 1659 s, 1613 s, 1539 m, 1461 w, 1429 m, 1362 m, 1172 m, 784 w, 746 w, 655 w, 623 w, 530 w, 470 w. mp = 171–174°C. Electrospray mass (masses given based on ^1^H, ^12^C, ^16^O, ^14^N, and ^120^Sn): the isotopic distributions were compared with the calculated. Only tin-containing fragments are given, *m/z*: [M^+^] ([(C_21_H_28_N_3_O_3_)Sn(CH_3_)_2_]^+^), 520 (100%); (3[M^+^] + CH_2_Cl_2_), 542 (16%); ([M^+^] + M), 1037 (7%).

*[(C*_21_ *H*_27_ *N*_3_ *O*_3_ *)Sn(CH*_2_ *CH*_2_ *CH*_2_ *CH*_3_)_2_ *](0.5CH*_2_ *Cl*_2_ *) (**2**)*. A yellow crystalline solid **2** was obtained in moderate yield (520 mg, 43.16%). Elemental analysis for C_29,5_H_46_ClN_3_O_3_Sn: Calcd(%): C, 54.94; N, 6.52; H, 7.19. Found (%): C, 54.70; N, 6.08; H, 7.32. IR (KBr disk) cm^−1^: 3447 m, 3276 m, 2958–2869 s, 1668 s, 1611 s, 1539 m, 1460–1426 m, 1363 m, 1172 m, 840 w, 746 w, 655 w, 622 w, 532 w, 480 w. mp = 110–116°C. Electrospray mass (masses given based on ^1^H, ^12^C, ^16^O, ^14^N, and ^120^Sn): the isotopic distributions were compared with the calculated. Only tin-containing fragments are given, *m/z*: [M^+^] ([(C_21_H_28_N_3_O_3_)Sn(CH_2_CH_2_CH_2_CH_3_)_2_]^+^), 604 (100%); (4[M^+^] + CH_2_Cl_2_), 625 (11%); ([M^+^] + M), 1205 (3%).

*[(C*_21_ *H*_27_ *N*_3_ *O*_3_ *)Sn(C*_6_ *H*_5_)_2_ *] (**3**)*. A yellow solid **3** was obtained in moderate yield (660 mg, 47.66%). Elemental analysis for C_33_H_37_N_3_O_3_Sn: Calcd(%): C, 61.70; N, 6.54; H, 5.81. Found(%): C, 61.59; N, 6.39; H, 5.21. IR (KBr disk) cm^−1^: 3448 m, 3306 m, 2957–2870 s, 1668 w, 1618 s, 1538 m, 1461 m, 1431 m, 1361 m, 1253 m, 1171 m, 837 w, 731 m, 654 w, 620 w, 536 w, 450 w. mp = 181–185°C. Electrospray mass (masses given based on ^1^H, ^12^C, ^16^O, ^14^N, and ^120^Sn): the isotopic distributions were compared with the calculated. Only tin-containing fragments are given, *m/z*: [M^+^] ([(C_21_H_28_N_3_O_3_)Sn(C_5_H_5_)_2_]^+^), 643 (100%); (4[M^+^] + CH_2_Cl_2_), 666 (12%); ([M^+^] + M), 1285 (6%).

*[(C*_21_ *H*_28_ *N*_3_ *O*_3_ *)Sn(CH*_2_ *CH*_2_ *CH*_2_ *CH*_3_)_3_ *] (**4**)*. A yellow crystalline solid **4** was obtained in moderate yield (410 mg, 31.03%). Elemental analysis for C_33_H_55_N_3_O_3_Sn: Calcd(%): C, 60.01; N, 6.36; H, 8.39. Found(%): C, 60.29; N, 6.46; H, 7.99. IR (KBr disk) cm^−1^: 3091 w br, 2955–2866 s, 1611 s, 1460–1439 m, 1366 s, 1269–1239 m, 1174 w, 818 s, 655 m, 450 w. mp = 200–203°C. Electrospray mass (masses given based on ^1^H, ^12^C, ^16^O, ^14^N, and ^120^Sn): the isotopic distributions were compared with the calculated. Only tin-containing fragments are given, *m/z*: [HSn(CH_2_CH_2_CH_2_CH_3_)_3_^+^], 291 (87%); [M^+^] ([(C_21_H_29_N_3_O_3_)Sn(CH_2_CH_2_CH_2_CH_3_)_3_]^+^), 662 (100%); ([M^+^] + Bu_3_Sn), 951 (41%).

*[(C*_21_ *H*_28_ *N*_3_ *O*_3_ *)Sn(C*_6_ *H*_5_)_3_ *](2.2CH*_2_ *Cl*_2_ *)(3.6C*_6_ *H*_5_ *CH*_3_ *) (**5**)*. A yellow solid **5** was obtained in moderate yield (380 mg, 15.52%). Elemental analysis for C_66.4_H_76.2_N_3_O_3_SnCl_4.4_ Calcd(%): C, 64.37; N, 3.39; H, 6.20. Found(%): C, 64.27; N, 3.35; H, 5.56. IR (KBr disk) cm^−1^: 3266–3060 m, 2958–2864 s, 1962–1716 w, 1631–1595 s, 1479 m, 1427 s, 1361 m, 1260 w, 1180 w, 1073 s, 729 (CH_2_Cl_2_) s, 697 (CH_2_Cl_2_) s, 657 m, 455 m, 443 m. mp = 203–205°C. Electrospray mass (masses given based on ^1^H, ^12^C, ^16^O, ^14^N, and ^120^Sn): the isotopic distributions were compared with the calculated. Only tin-containing fragments are given, *m/z*: [M^+^] ([(C_21_H_29_N_3_O_3_)Sn(C_6_H_5_)_3_]^+^), 722 (23%); [C_21_H_29_N_3_O_3_]^+^, 371 (22%); [C_6_H_5_]^+^, 77 (100%).

#### 2.1.2. Biological Activity: Antibacterial Screening

*Bacterial Strains*. Bacterial reference cultures and self-collection strains were used to test a broad spectrum of bacteria. The strains used in this study were Enterohemorrhagic *Escherichia coli* EDL933, *Staphylococcus aureus* MRSA, *Pseudomona aeruginosa* ATCC27853, *Shigella dysenteriae* FMUNAM98863, *Salmonella typhimurium* ATCC14028, *Salmonella typhi* FMUNAM95073, *Proteus mirabilis* RTX339, *Klebsiella pneumoniae* ATCC8045, and *Serratia marcescens* TSUAM1.

Isolates of methicillin-resistant *S. aureus* (MRSA) were obtained from our collection [16]. Identification of *S. aureus* was performed by the coagulase test with human plasma. MRSA strains were confirmed to be methicillin resistant by testing for methicillin susceptibility and by PCR detection of the *mecA* gene.

The organotin compounds were tested *in vitro* for the antibacterial activity against clinical pathogen and reference strains using the disk diffusion test, in accordance with the procedures outlined by CLSI (formerly the NCCLS) [17]. Muller-Hinton agar was used for this method. Inocula were adjusted to 1.5 × 10^8^ CFU mL (0.5 McFarland). BD BBLTM Sensi-Discs (Becton Dickinson and Company, Sparks, MD, USA) were used as growth inhibition controls. The antibiotics tested were Kanamycin (30 *μ*g), Vancomycin (30 *μ*g), Chloranphenicol (30 *μ*g), Cloxacillin (1 *μ*g), and Penicillin (10 IU). 

The degree of effectiveness was measured by determining the diameters of the zone of inhibition caused by the compound. Compounds (**1**–**5**) were used, each one with a concentration of 6 mg per disc. The plates were incubated at 37°C for 24 h. During incubation time the complexes diffused from the disc to the medium. Effectiveness was classified into four different categories based on diameter of the zone of inhibition: (+++) most effective, (++) moderate effective, (+) slightly effective, and (−) noneffective. The results were compared against those of controls, which were screened simultaneously. The determinations were performed by triplicate and the results are reported as average values.

## 3. Results and Discussion

### 3.1. Synthesis

**Figure 1:**
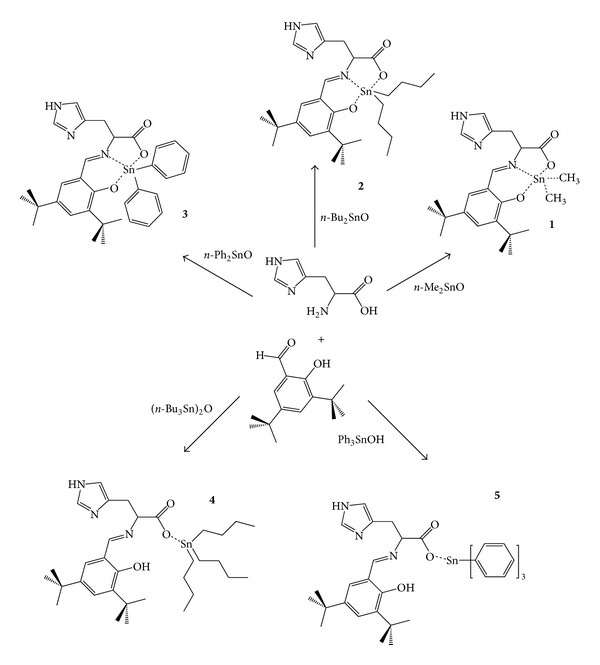
Schematic representation of compounds **1**–**5** and their synthetic procedure.

Compounds **1**–**5** are prepared by *in situ* condensation of L-histidine with 3,5-di-*tert*-butyl-2-hydroxybenzaldehyde in the presence of the corresponding tin(IV) derivatives: di-*n*-methyltin oxide, di-*n*-butyltin oxide, di-*n*-phenyltin oxide, bis-tri-*n*-butyltin oxide, or triphenyltin hydroxide. These conditions affording compounds **1**, **2**, **3**, **4**, and **5**, respectively, and the proposed structures are shown in Figure 1. In all complexes, the Schiff base histidine derivative is coordinated to the alkyl- or aryl-tin(IV) ion through the oxygen atom belonging to the carboxylate group. Compounds **1**, **2**, and **3** are also coordinated to the Schiff derivative through the imino nitrogen and the phenolic oxygen in order to stabilize a 5-coordinated tin(IV) core. In compounds **4** and **5**, tin is 4-coordinated.

All complexes are light yellow crystalline solids which are stable in air. Most of them are soluble in organic solvents. Compound **5** shows a very limited solubility in ethanol. Empirical formulae and proposed structures for all the compounds are confirmed by analytical and spectral data which are described in the following lines.

### 3.2. Infrared Spectroscopy

The coordination sphere on compounds **1**–**5** has been analyzed by means of FT-IR. The binding mode of the L-histidine Schiff base derivative to metal in the complexes has been studied by comparison of IR spectra of free ligand precursors and metal complexes. The absence of the broad O–H stretching frequencies in the region 3170–2870 cm^−1^ denotes that deprotonation and coordination of the carboxylate group to tin have taken part during the complex formation in all compounds. 

Free L-histidine shows a strong band at 1630 cm^−1^, which is assigned to the stretching vibration of the C=O bond. There are also medium strength bands at 1589–1571 cm^−1^ that are assigned to the C=N and C=C stretching vibration. The corresponding bands in the tin compounds are shifted to higher frequencies as expected, due to tin coordination. For pentacoordinated compounds **1**, **2**, and **3**, the observed shifts are in the range 40–30 cm^−1^. For tetracoordinated tin compounds **4** and **5**, the shifts are smaller in the range 11–2 cm^−1^. This observation is compatible with some other 4-coordinated structures described in the literature [9, 18]. Moreover, the azomethine band is easily observed at 1539–1538 cm^−1^ region for compounds **1**, **2**, and **3**. This red shift is considered the result of coordination of this moiety to the metal center (see Figure 1) [6]. For compounds **4** and **5**, this *ν*(C=N) band is not observed in such region, evidence that demonstrates that the azomethine moiety is not involved in coordination to tin.

The carboxylate moiety in compounds **1**–**5** has been structurally characterized by the intense and broad absorptions in the range 1660–1330 cm^−1^, due to asymmetric and symmetric stretching modes. The difference between asymmetric and symmetric O–C=O stretching vibrations (Δ*ν*) has been used to determine the mode of coordination of a carboxylate ligand with metals [9, 19, 20]. Differences larger than 250 cm^−1^ are indicative of monodentate carboxylate complexes, while Δ*ν* values in the range 150–250 cm^−1^ are indicative of compounds with carboxylate-bridged structures. Finally, it is presumed that a chelation is present, if this parameter is lower than 150 cm^−1^. 

The Δ*ν* = *ν*_as_(OCO) − *ν*_s_(OCO) for the proposed pentacoordinated compounds **1**, **2**, and **3** is the following: Δ*ν* = 297, 305, and 307 cm^−1^, respectively. For the tetracoordinated compounds **4** and **5**, Δ*ν* is Δ*ν* = 245 and 270 cm^−1^, respectively. All values of Δ*ν* for compounds **1**–**5** are in the range 310–245 cm^−1^, and this evidence strongly suggests that the carboxylate group in all compounds adopted a unidentate nature.

Some other stretching frequencies of interest are those characteristics of the *ν*(Sn–C), *ν*(Sn–O), and *ν*(Sn–N) bonds frequencies. All these values are consistent with those detected in a number of organotin(IV)-oxygen, organotin(IV)-carbon, and organotin(IV)-nitrogen derivatives [10, 21, 22]. Worthy mentioning is the presence of two *ν*(Sn–O) weak bond frequencies for compounds **1** (655, 623 cm^−1^), **2** (655, 622 cm^−1^), and **3** (654, 620 cm^−1^) [23]. This evidence could be explained in views of two different Sn–O bonds. Compounds **4** and **5** only show one stretching Sn–O vibration. Then it suggests that just one type of Sn–O bond is observed, confirming the tetracoordinated sphere for tin in the compounds. 

The recorded high-resolution mass spectra of **1**–**5** and molecular ion peaks are in agreement with the proposed structures. The MS peaks present the correct isotopomer distribution, as expected for the tin isotope distribution.

**Figure 2:**
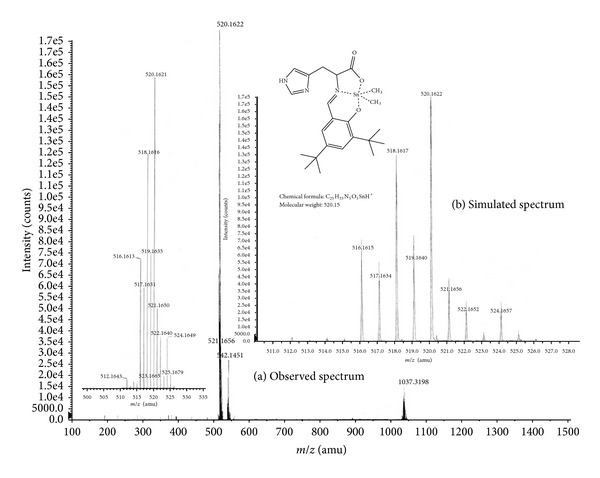
Observed mass spectrum (a) and simulated spectrum (b) of complex **1**.

**Figure 3:**
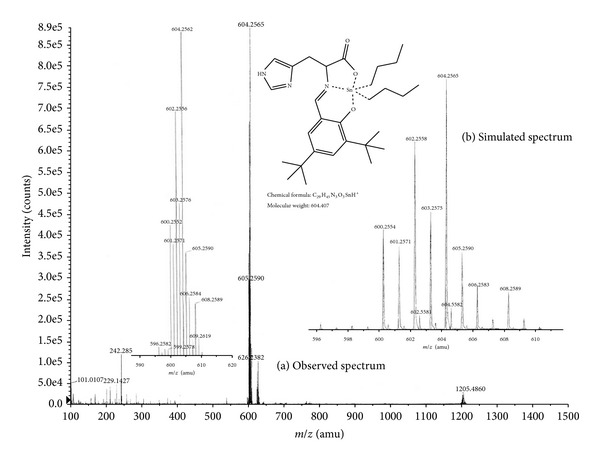
Observed mass spectrum (a) and simulated spectrum (b) of complex **2**.

For instance, in complex **1**, the degradation pattern shows peaks at 520 (100% *m/z*) that indicates molecular ion peak [M^+^, [(C_21_H_28_N_3_O_3_)Sn(CH_3_)_2_]^+^], whereas peaks at 542 (16% *m/z*) and 1037 (7% *m/z*) show 3[M^+^] + CH_2_Cl_2_ and [M^+^] + M, respectively (Figure 2). Complex **2** shows the normal trends of degradation patterns (Figure 3). The various degradation peaks at 604 (100% * m/z*) show molecular ion; the peaks at 625 (11% *m/z*) show 4[M^+^] + CH_2_Cl_2_; peaks at 1205 (3% *m/z*) show [M^+^] + M.

Complexes **3**–**5** again show a similar pattern of degradation.

### 3.3. Multinuclear NMR Spectroscopy

The ^1^H, ^13^C, and ^119^Sn NMR spectra of compounds **1**–**4** have been recorded in CDCl_3_ or MeOD and the assignments obtained through 1D and 2D experiments. When necessary, ^119^Sn NMR spectra have also been recorded in the solid state. The very limited solubility of compound **5** prompted us to obtain the ^13^C and ^119^Sn spectra in the solid state only.

The ^1^H NMR spectra assignment of compounds **1**–**5** is based on integration values, chemical shifts, coupling constants, and ^1^H-^13^C HMQC experiments. Starting and model compounds spectra were also useful for comparison reasons [18]. The expected resonances are observed for L-histidine, 3,5-di-*tert*-butyl-2-hydroxybenzaldehyde, tributyltin(IV), triphenyltin(IV), dibutyltin(IV), dimethyltin(IV), and dibutyltin(IV) moieties. Due to rapid exchange between the two nitrogen atoms of the imidazole ring and probably with deuterium from solvent, the N–H proton from the imidazole moiety is not observed in the spectra. 

**Figure 4:**
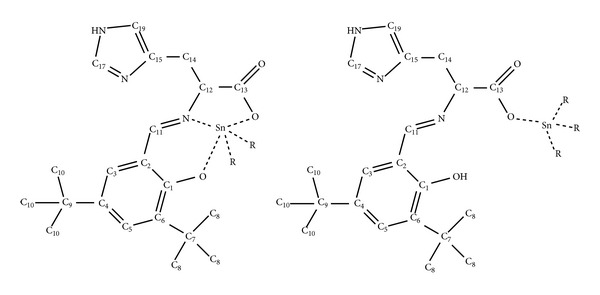
Numbering system used in pentacoordinated and tetracoordinated tin(IV) compounds for the NMR spectra assignment. R = CH_3_–, CH_3_CH_2_CH_2_CH_2_– (CH*α*, CH*β*, CH*γ*, CH*δ*) or C_6_H_5_– (CHo, CHm, CHp).

The results obtained are listed in Tables 1 and 2. For comparative purposes the data are tabulated using a general numbering scheme (Figure 4).

The characteristic resonance peak in the ^1^H NMR spectra of compounds **1**–**5**, at 8.4–7.9 ppm, reveals the presence of the imino moiety H–C11=N, which confirms the condensation of the L-histidine derivative (Figure 1). Additionally, for compounds **1**, **2**, and **3**, coordination to Sn(IV) is confirmed by small coupling peaks associated to the H–C11=N resonance peak. This spin-spin coupling between the azomethine proton and the tin nucleus, ^3^ *J*(Sn–N=CH), presents coupling constants in the range 52–70 ppm, values that are in agreement with a magnetic coupling between the tin nuclei located in a transposition with respect to the azomethine proton [23, 24]. In complex **1**, characteristic peaks of the methyl groups attached to the tin atom are observed at 0.64 and 0.56 ppm as expected [25]. In complex **2**, the presence of the Sn–Bu_2_ resonance peaks, H*α*, H*β*, H*γ*, and H*δ*, confirms the chemical nature of the dibutyl moiety of **2**. In compound **4**, the butyl protons appear in the range 0.80–1.53 cm^−1^, with multiplicity and integration values in agreement with the proposed structure. For complex **3** the signals corresponding to the phenyl groups are clearly observed.

The ^1^H NMR spectra of all the complexes present a resonance peak assigned to H–C12 moiety. This moiety is attached to the carboxylic group and it is observed in the range 4.14–4.59 ppm. These are clearly downfield shifted when comparing with the resonance peak in the free ligand at around 3.98 ppm (D_2_O). This evidence suggests that the downfield effect is due to coordination to tin. In this order of ideas, the ^1^H NMR spectra of all complexes present a resonance for H–C14 with the same downfield shift effect (3.15–3.47), with a minimal shift for compounds **3** and **4**. The signals corresponding to the *tert*-butyl protons of the ligand have also been assigned and all data is presented in Table 1.

Table 2 presents the ^13^C NMR and ^119^Sn NMR data for the synthesized organotin compounds following the same numbering scheme presented on Figure 4. The very limited solubility of compound **5** prompted us to obtain the ^13^C and ^119^Sn spectra in the solid state. The ^13^C NMR characterization was obtained through comparison with starting materials, and structural related analogues. 

The ^13^C NMR data explicitly resolved the resonances of all the distinct carbon atoms present in all the complexes. The ^13^C NMR data for all compounds show that the resonance peak of the carboxyl carbon, C13, appears in the range from 173 to 177 ppm, in agreement with the data reported for analogous derivatives [18]. The signal of the imine carbon, C11, appears in the 166–177 ppm range, showing in some cases a marked deshielding effect with respect to the imine group. The Sn–N coordination should produce an N–C polarization. The biggest polarization effect could be observed, as expected, in compound **3** where phenyl groups are attached to tin. 

For compounds **4** and **5**, the signal of the imine carbon, C11, appears in the range 165–167 ppm, which is consistent with the lack of the Sn–N coordination. The same high field shift effect could be observed in case of C2.

The ^1^ *J*(^13^C–^117/119^Sn) coupling constant values are of major importance in the estimation of the coordination mode of the tin atom. The values of ^1^ *J*(in solution) can be related to the tin-coordination number. Thus these values are a qualitative evidence of the organotin(IV) compound structure [26–28]. Pentacoordinated organotin(IV) ions show ^1^ *J* values between 450–610 Hz, while tetracoordinated organotin(IV) ions show values in the range 325–440 Hz [18]. 

The ^1^ *J* coupling constants for compounds **1** and **2** suggest a pentacoordinated nature in solution. For compound **3** the ^1^ *J* coupling constants are not observed. For compound **4** the ^1^ *J* values are 415 and 435 Hz, suggesting a four-coordinated structure in solution. The ^2^ *J*(^13^C–^117/119^Sn) = 72/70 Hz also suggests a tetrahedral geometry for **4**.

The correct assignment of ^13^C NMR resonances of the *n*-butyl group in **2** is determined by the coupling constants ^*n*^ *J* (^13^C–^117/119^Sn). Compound **2** displays a resonance peak for the *n*-butyl C*α* at 21-22 ppm with two very close coupling signals. This observation provides additional evidence for the magnetic equivalence of the butyl moieties. The same criterion is followed in the assignment of ^13^C NMR resonances of the *n*-butyl group in **4**. The magnetic equivalence of the butyl moieties is concluded from the spectra.

In order to provide further evidence for the proposed structures of the complexes in solution, ^119^Sn NMR spectra are analyzed. The ^119^Sn chemical shifts are extremely dependent on the coordination number for tin and the nature of the groups directly coordinated to the metal. The chemical shift values for compounds **1**–**3** are within the expected range for pentacoordinated tin ions with a single resonance peak in solution. There are not changes in the solid state spectra as could be appreciated by the chemical shifts observed in the corresponding solid ^119^Sn NMR spectra. Thus, the structural composition in the solid state is retained in solution for compounds **1**, **2**, and **3**.

^119^Sn NMR spectrum of compound **4** exhibits a single resonance in solution, with a chemical shift of 39.44 ppm. In the solid state, the chemical shift observed is characteristic of a five-coordinated compound. This evidence suggests that in the solid state inter- or intramolecular interactions could be responsible of the increase in coordination number. 

No ^119^Sn resonance could be observed for compound **5** in solution, due to its limited solubility. In addition the chemical shift observed in the ^119^Sn NMR spectrum in the solid state suggests the tetracoordinated nature of **5**. Despite the Lewis acidity that a phenyl group could induce in the tin atom [29], this is very low as this compound appears unable to extent its coordination sphere. 

### 3.4. Microbial Activities

Organotin(IV) cations with biological active ligands are a major research debt for the scientific efforts in the search of new metal-based drugs, but little work is available on the bactericidal properties of organotin(IV) derivatives of amino acids in the literature. In particular, organotin derivatives of amino acids containing N-heterocycles bonded to the *α*-carbon should be studied in more detail because of the well-known role of the histidine residue binding properties. In particular it is important to study the histidine binding properties to triorganotin compounds in order to provide evidence for important mechanisms of biological activities [30]. 

In order to study the potential biological properties of the synthesized compounds, the bactericidal properties are studied. 

The degree of effectiveness was measured by determining the diameters of the zone of inhibition caused by the compound. The biological activity was then classified in 4 categories on the bases of their diameter of growth inhibition: (+++) most effective, (++) moderate effective, (+) slightly effective, and (−) noneffective. 

Compound **1** is slightly effective against both Gram-positive and Gram-negative bacteria; **2** is moderately effective against Gram-negative bacteria; **3** does not show antibacterial activity at all. These results are comparable to related pentacoordinated tin(IV) compounds with Schiff bases derived from amino acids reported in the literature [2].

**Figure 5:**
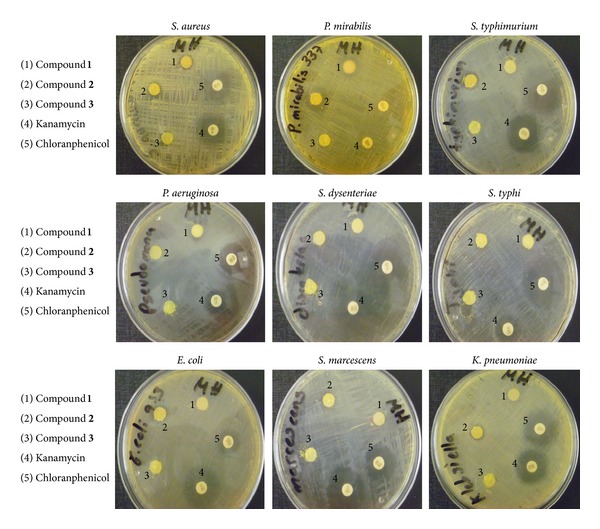
Photographs of the antimicrobial activities of compounds **1**, **2**, and **3** against *S. aureus*, *P. mirabilis*, *S. typhimurium*, *P. aeruginosa*, *S. dysenteriae*, and *S. typhi*, showing their inhibition zone.

**Figure 6:**
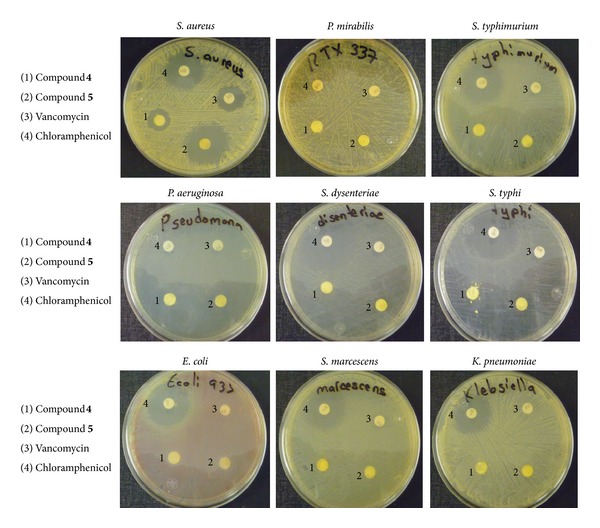
Photographs of the antimicrobial activities of compounds **4** and **5** against *S. aureus*, *P. mirabilis*, *S. typhimurium*, *P. aeruginosa*, *S. dysenteriae*, and *S. typhi*, showing their inhibition zone.

Compounds **4** and **5** are moderate effective and most effective, respectively, against *Staphylococcus aureus* strains (Gram-positive). Results are presented in Table 3 and Figures 5 and 6.

Thus the results clearly indicate that pentacoordinated tin(IV) compounds of Schiff bases derived from L-histidine have exhibited moderate to minimal bactericidal properties while tetracoordinated tin(IV) compounds have presented high activity against the Gram-positive methicillin-resistant *S. aureus*. Even more, compound **5** as observed in Figure 6 is more active when compared with standard references vancomycin and chloramphenicol against the same bacteria under identical experimental conditions. These are very promising results in particular for the triphenyltin(IV) derivative. 

It has been described that triorganotin compounds are significantly more biocidally active than other classes [30], statement that is true based on the results of this work.

## 4. Conclusions

Five novel tin(IV) compounds have been synthesized. The compounds are coordinated to a histidine analogue. With the help of various physicochemical techniques, the structure of the newly synthesized compounds has been proposed (Figure 1). It has been spectroscopically demonstrated that the Schiff base ligand derived from L-histidine and 3,5-di-*tert*-butyl-2-hydroxybenzaldehyde coordinates to tin(IV) in a tridentate and monodentate manner producing pentacoordinated and tetracoordinated tin(IV) compounds. 

Amino acids are very difficult ligands to work with due to their many donor groups and reduced solubility in nonpolar solvents. Nevertheless it was possible to isolate pure compounds. The selection of the ligand, a biologically active amino acid, has produced metal-ligand combined effects that may result in an important improvement in the activity of the resulting coordination compounds.

The triphenyltin(IV) complex **5** has been found to be most active against the Gram-positive methicillin-resistant *S. aureus*.

With these results, it is important to stress the need of more research in the synthesis, isolation, and characterization of new organotin(IV) cations with biologically active ligands, in an effort to obtain important structure-activity relationships. 

## Figures and Tables

**Table 1:** ^
1^H NMR data of compounds **1**–**5**.

Compound numbering	**1** CDCl_3_	**2** CDCl_3_	**3** CD_3_OD	**4** CDCl_3_
H11	8.13 (s, 1H) [58]	7.91 (s, 1H) [52]	8.33 (s, 1H) [70]	8.34 (s, 1H)
H17	7.63 (s, 1H)	7.60 (s, 1H)	7.08 (s, 1H)	7.48 (s, 1H)
H5	7.54 (d, 1H) [2.4]	7.51 (d, 1H) [2.7]	7.63 (d, 1H) [2.4]	7.35 (d, 1H) [2.4]
H3	6.85 (d, 1H) [2.4]	6.76 (d, 1H) [2.7]	7.07 (d, 1H) [2.5]	7.03 (d, 1H) [2.4]
H19	6.80 (s, 1H)	6.73 (s, 1H)	6.52 (s, 1H)	6.73 (s, 1H)
H12	4.43 (m, 1H)	4.43 (m, 1H)	4.59 (m, 1H)	4.14 (m, 1H)
H14	3.39–3.25 (m, 2H)	3.47–3.44 3.11–3.07 (m, 2H)	3.27–3.16 (m, 2H)	3.31–3.15 (m, 2H)
H*γ*		1.73–1.51 (m, 4H)		1.27–1.28 (m, 6H)
H*α*		1.4–1.3 (m, 4H)		1.27–1.28 (m, 6H)
H*β*		1.4–1.3 (m, 4H)		1.53 (m, 6H)
H*δ*		0.92 (t, 3H) [7.3] 0.79 (t, 3H) [7.3]		0.80 (t, 9H) [7.3]
Ho			7.87–7.82 (m, 4H)	
Hm			7.58–7.29 (m, 4H)	
Hp			7.58–7.29 (m, 4H)	
H8, H10 t-butyl CH_3_	1.37 (s, 9H) 1.28 (s, 9H)	1.37 (s, 9H) 1.25 (s, 9H)	1.47 (s, 9H) 1.30 (s, 9H)	1.41 (s, 9H) 1.28 (s, 9H)
CH_3_-Sn	0.64 (s, 6H), 0.56 (s, 6H)			

Data obtained at 500 MHz. Chemical shifts in ppm with respect to TMS; coupling constants in hertz, ^*n*^ *J*(^1^H–^1^H) and ^*n*^ *J*(^1^H–^119/117^Sn) between brackets.

s: singlet; d: doublet; t: triplet; m: complex pattern. Assignment base in 1D and 2D NMR studies.

**Table 2:** ^
13^C and ^119^Sn NMR data of compounds **1**–**5**.

Compound numbering	**1** CDCl_3_	**2** CDCl_3_	**3** CD_3_OD	**4** CDCl_3_	**5** Solid state
C11	174.33	174.54	177.66	167.57	166.6
C13	173.72	173.72	175.79	175.62	176.6
C2	166.59	167.0	168.61	158.13	157.55
C1	141.29	140.96	143.61	140.00	a
C4	139.04	138.64	142.00	136.67	a
C6	135.70	135.67	134.63	134.09	a
C5	133.19	133.08	132.97	127.16	a
C17	131.08 (b)	131.30 (b)	132.64 (b)	131.98 (b)	—
C3	129.49	129.47	129.95	126.24	a
C19	119.78 (b)	119.84 (b)	118.00 (b)	123.69 (b)	120.2
C15	116.61	116.51	119.66	117.98	117.5
C12	67.18	67.42	68.56	72.02	64.0
C7	35.27	35.19	35.45	35.08	34.68
C9	34.06	33.93	34.17	34.16	33.86
C14	33.97	33.90 (b)	33.98	29.50 (b)	29.5
C8	31.23	31.10	30.56	31.54	31.0
C10	29.44	29.36	29.47	29.50	29.2
CH_3_	1.23, 0.933 [653/648]				
C_*β*_		27.00 [38] 26.85 [n.o.]		28.07 [24]	
C_*γ*_		26.74 [91] 26.56 [92]		27.02 [72/70]	
C_*α*_		22.26 [599] 22.20 [599]		17.73 [435/415]	
C_*δ*_		13.59 13.39		13.66	
C_i_			142.67 [n.o.] 141.27 [n.o.]		a
C_o_			137.78 [53] 137.32 [53]		a
C_m_			130.54 [87] 130.23 [83]		a
C_p_			132.12 [n.o.] 131.64 [16]		a
^ 119^Sn	−162.16 CDCl_3_	−197.58 CDCl_3_	−369 CD_3_OD	39.44 CDCl_3_	No soluble
^ 119^Sn Solid state	−169.50	−196.68	−455.87	−112.59	−122.09

Chemical shifts in ppm with respect to TMS; ^119^Sn chemical shifts in ppm with respect to (CH_3_)_4_Sn; ^*n*^ *J*(^13^C–^119/117^Sn) coupling constants between square brackets. n.o.: not observed.

a: Unambiguous assignment of this resonance peak was not reached because there are multiple broad signals included in the range 143–127 ppm.

**Table 3:** Antimicrobial activity of organotin(IV) compounds **1**–**5**.

Strain	Compound				
	**1**	**2**	**3**	**4**	**5**
*Staphylococcus aureus *	+	−	−	++	+++
*Proteus mirabilis *RTX337	+	+	−	−	−
*Pseudomona aeruginosa *	+	−	−	−	−
*Salmonella typhi *	−	+	−	−	−
*Salmonella typhimurium *	−	+	−	−	−
*Escherichia coli *933	−	+	−	−	−
*Klebsiella *	−	+	−	−	−
*Serratia marcescens *	−	−	−	−	−
*Shigella dysenteriae *	−	−	−	−	+
